# Multimodal interface and reliability displays: Effect on attention, mode awareness, and trust in partially automated vehicles

**DOI:** 10.3389/fpsyg.2023.1107847

**Published:** 2023-02-28

**Authors:** Noé Monsaingeon, Loïc Caroux, Sabine Langlois, Céline Lemercier

**Affiliations:** ^1^Cognition, Language, Languages and Ergonomics Laboratory (CLLE), University of Toulouse and CNRS, Toulouse, France; ^2^Renault Technocentre, Guyancourt, France

**Keywords:** automated driving, mode confusion, uncertainty, earcon, haptic, peripheral vision

## Abstract

The goal of this study is to evaluate the effect of a multimodal interface indicating the limits of automation in order to stimulate an appropriate level of attention and to induce accurate mode awareness and trust in partial driving automation. Participants drove in a driving simulator with partial driving automation and were confronted with surprising situations of suspension of driving automation systems in different contexts. They drove the simulator during three driving sessions, with either a multimodal interface indicating the limits of automation or a visual basic interface. Their driving performance, ocular behavior, and subjective evaluation of trust and workload were evaluated. The results revealed that the multimodal interface stimulates an appropriate level of attention and increases mode awareness and trust in automation, but these effects are context-dependent. The indications of the limits of automation improved the knowledge regarding automation, but this knowledge did not necessarily lead to improved driving performance. Design solutions are discussed to support the improvement of driving performance for take-overs in vehicles equipped with partial driving automation.

## 1. Introduction

Partial driving automation can supervise lateral and longitudinal controls of a vehicle through a combined function approach (NHTSA, 2013). The combination of Lane Centering Assist (LCA) and Adaptive Cruise Control (ACC) allows the vehicle to be automatically centered in its lane, with its speed matched with a followed vehicle. The Society of Automotive Engineers (SAE, 2021) considers these two coordinated systems as the second level of driving automation system, out of five levels of automation. In SAE Level 2, referenced here as Partial Driving Automation (PDA), driving automation systems control the vehicle in specific conditions, while the human operator is responsible for monitoring the activity of driving automation systems. In situations of sharp bends, the LCA can reach its limits and transfer steering control back to the driver, while the speed is still regulated automatically. In these limited situations, the drivers need to be prepared to control the vehicle and be aware of the state of driving automation system to know which part of the driving activity (lateral, longitudinal, or both) they need to take over. Through the interface, the drivers perceive and interpret the functioning of automations, allowing them to form a mental model (i.e., a representation of a system’s purpose, form, functioning, state, and structure; Seppelt and Victor, 2020) and calibrate trust in it (i.e., their attitude toward an agent that helps them achieve a goal in a situation where uncertainty and vulnerability are involved; Lee and See, 2004). Previous studies revealed that interfaces transmitting signals through multiple sensory channels (i.e., a multimodal interface) and displaying the limits of automation allow to improve the interaction with driving automation systems (Beller et al., 2013; Zhang et al., 2019). It was proposed by previous authors that improving knowledge about automation’s limits leads to improving trust in it (Seppelt and Lee, 2019). Several usages are needed for drivers to form efficient knowledge about the functioning of the automation (Forster et al., 2019). This study proposed to investigate the effect of prolonged usage of a multimodal interface indicating the limits of automation on the allocation of attentional resources, the identification of modes of automation, the quality of mental models, and trust in driving automation systems.

### 1.1. Challenges of automated driving

Goals and guidelines for interfaces of automated vehicles were established by Carsten and Martens (2019) to respond to the main challenges raised by automated driving. Those goals are based on Rasmussen’s (1983) Skills Rules Knowledge model of performance of skilled human operators. Following ecological interface design principles, the interfaces should aim to induce behavior with fast cognitive processing instead of slower higher cognitive knowledge processing. In line with Carsten and Martens’ work, the present study evaluates the effect of interfaces on the following challenges: (1) providing required understanding of the automated vehicles capabilities and status (minimize mode errors); (2) engendering correct calibration of trust; (3) stimulating appropriate level of attention and intervention; and (4) minimizing automation surprises. The goal (1) aims to ensure that drivers identify the status of automation, which is usually done by the interface: “a visual interface is typically presented in current commercially available systems. The most basic feedback a system can provide is showing whether the system or a function is activated or not” (Carsten and Martens, 2019, p. 6). The goal (4) aims to ensure that the interface avoids causing automation surprises, described as “situations where the driver actually notices the action is inconsistent with drivers expectation” (Carsten and Martens, 2019, p. 11). The situations described here refer to a detachment between the drivers’ excepted driving automation system’s status and the actual one. Therefore, the goal (1) aims to provide information that enables the identification of modes of driving automation system, and the goal (4) avoids the occurrence of errors related to the identification of the status of the driving automation system. Mode awareness, defined here as awareness of the current mode of driving automation system and the knowledge encompassing the existing modes (Kurpiers et al., 2020), allows to describe the identification of modes and the likely occurrence of automation surprises. An accurate mode awareness implies that modes have been identified and, therefore, automation surprises are not expected to occur. Therefore, we propose to gather goals (1) and (4) under a common global goal of increasing mode awareness. The three goals are discussed separately in three different paragraphs before proposing interface solutions.

#### 1.1.1. Attention allocation

Humans have difficulties maintaining visual attention on a monitoring task in which less is happening for long periods of time (Bainbridge, 1983). When PDA is activated, the drivers must effectively monitor the lateral and longitudinal positioning of the vehicle to be ready to take over when a limit of automation is reached. When a limit of automation is reached, a suspension of driving automation occurs, meaning that the driving automation systems initiate a transition of control from automation to the drivers. A suspension of automation requires a switch of attention from monitoring to controlling the vehicle (Carsten and Martens, 2019). When the monitoring task to be performed is easy, long-lasting, and monotonous, the individuals tend to wander in their thoughts (Lemercier et al., 2014; Berthié et al., 2015). The out-of-the-loop phenomenon, defined here as a temporary disengagement from the control and the monitoring loops (Carsten and Martens, 2019), can be observed in such configurations. To avoid the out-of-the-loop phenomenon leading to misses or false alarms of suspensions, interfaces of automated vehicles should allow allocating attention efficiently, facilitating the switch from monitoring to control. By doing so, drivers should be able to identify the status of automation and, therefore, improve their mode awareness.

#### 1.1.2. Mode awareness

Mode awareness is founded on two dimensions, namely, knowledge about automation’s functioning and awareness at a specific moment of the state of driving automation systems (Kurpiers et al., 2020). Experience with the driving automation system (i.e., the time spent using the system) plays a major role in correctly understanding the role one has to play in the interaction (Solís-Marcos et al., 2018). Interactions with an automated driving system enable the forging of a representation of its purpose, form, functioning, state, and structure, which can be merged into the term *mental model* (Seppelt and Victor, 2020). The more the users interact with an automated driving system, the more accurate their mental model will be (Beggiato et al., 2015; Forster et al., 2019). Therefore, longitudinal studies are necessary to capture the evolution of mental models. In addition, the quality of mental models can be influenced by the design of interfaces in vehicles equipped with driving automation systems (Seppelt and Lee, 2019). The quality of these mental models, therefore, depends on the quality of the information perceived and understood. If the information on the interface is misunderstood, an inaccurate mental model may develop. Consequently, a proper awareness of the status of the modes of automation is necessary to form an accurate mental model.

Regarding awareness of the status of driving automation systems, it is supported by the correct perception, comprehension, and capacity to project the future state of automation based on the available information (Endsley, 1995). This dimension relies mainly on the ability of drivers to perceive and understand interface signals about the status of driving automation systems, which is usually done through visual displays. As proposed by Kurpiers et al. (2020), an assessment of mode awareness can be performed by measuring three dimensions, namely, the drivers’ driving behavior, their ocular behavior, and their mental models. The behavior of the driver should be adapted to the mode of automation. When switching to manual driving, Deviation from Central Lane (DCL) or Time Headway (TH) should reveal that the drivers are in control of the vehicle. The ocular behavior of the drivers should reveal that their gaze is fixed on pertinent information for the automations’ status (e.g., the cluster before a potential suspension of automation). The mental models, evaluated through questionnaires regarding the functioning of automation depending on the situation, should be accurate. Interfaces indicating the status of PDA should allow drivers to form an accurate mental model and to be aware of the state of automation. With accurate knowledge about a system’s functioning, an appropriate degree of trust can be placed.

#### 1.1.3. Trust in automation

Trust in automation is impacted by the way the drivers perceive and understand its functioning. If automation does not accomplish the goals that it is intended to achieve (e.g., achieve the driving task), breakdown of trust can be observed (Parasuraman and Riley, 1997). With highly automated vehicles, drivers over trusting the driving automation system gazed less at the road (de Winter et al., 2014) or failed to take over correctly when needed (Flemisch et al., 2014). Therefore, trust in automation should be appropriately calibrated to the driving automation system’s capacity and limits. Informing the drivers of the driving automation systems’ capacity and reliability allows them to place adequate trust in it (Helldin et al., 2013). Trust that drivers have in driving automation systems is correlated with attention allocation (de Winter et al., 2014). It was proposed by previous authors that improving knowledge about automation’s limits leads to improving trust in automation (Seppelt and Lee, 2019). The influence of interface design on the long-term construction of mental models is yet to be investigated. It can be expected that multimodal interfaces indicating the limits of automation allow to form accurate mental models faster than classical visual-only interfaces.

### 1.2. Multimodal interfaces and reliability information

To address the three challenges mentioned above, the following two types of interfaces were investigated in this study: multimodal interface and automation reliability interface. Multimodal interfaces use multiple sensory modalities to convey information. They allow the attentional demands of the interface to be distributed across the multiple sensory channels, thereby reducing cognitive load compared to a situation where all demands are directed to a single sensory channel (Wickens, 2008). Earcons (i.e., abstract sounds representing meaning) indicating transitions of control from the system to the driver allow them to re-engage with the driving task (Petermeijer et al., 2017). Haptic feedback, in the form of kinesthetic and tactile feedback in the steering wheel, induces quick responses and is easily understood (Murata and Kuroda, 2015). Kinesthetic feedback can take the form of increased stiffness in the steering wheel, and tactile feedback can take the form of vibrations in the steering wheel (Gaffary and Lécuyer, 2018). Haptic feedback should reduce the risk of mode errors by informing the driver of the state of automation efficiently. Altogether, multimodal interfaces should induce more appropriate repartition of visual attention and make the identification of modes more accessible, allowing to respond to the first two challenges posed by automation: stimulate appropriate level of attention and induce accurate mode awareness. In addition to multimodal interfaces, information regarding the reliability of automation should allow the drivers to anticipate transitions of modes.

Reliability interfaces indicating the proximity to limits of automation aim to enable drivers to anticipate take-overs. Displaying automation’s uncertainty through gradual displays, pulsing LEDs, or varying colors in the cluster has led to faster reaction take-overs and better anticipation of automation suspensions (Beller et al., 2013; Helldin et al., 2013; Kunze et al., 2019; Monsaingeon et al., 2020). An Indicator of Proximity to the Limits of Automation (IPLA) is proposed in this article and aims at exploiting information already present in the vehicle to anticipate suspensions of automation. Indeed, digital information about the environment is currently used to operate the driving automation systems (e.g., lateral acceleration, current speed, GPS position). One operating limit of PDA is the lateral acceleration of the vehicle during bends, which is itself dependent on the radius of a bend and the speed. Some vehicles use environmental mapping to make ACC more comfortable by adapting the vehicle’s speed to a sharp bend.^1^ This type of information can also be used to prevent future suspensions of automated driving systems by projecting the future lateral acceleration based on the radius of the upcoming bend and the current speed of the vehicle. The idea of the IPLA is to exploit this information and transfer it to an indicator for the driver. An indicator of this nature presented in peripheral vision should allow the drivers to direct their attentional resources to the road when control is needed, addressing the first challenge of automation, i.e., stimulate appropriate attention. However, the addition of visual information can capture attention and increase mental workload (Monsaingeon et al., 2019). It can be expected that with training to use the interface, the mental workload would decrease (Christoffersen et al., 1996). By informing on the limits of automation, drivers should be able to anticipate transitions of modes and learn to identify situations in which these transitions may occur, thus addressing the second challenge of automation, i.e., induce accurate mode awareness. Finally, by informing on the situations that automation can or cannot handle, drivers should calibrate their trust accordingly, addressing to the third challenge of automation.

### 1.3. Methodology overview

The experimental method of this study aimed to assess the longitudinal effect of interface modalities on attention allocation, mode awareness, and trust in automation while interacting with partial driving automation systems. Participants were recruited following specific criteria. They were assigned to either one of two interface conditions. The participants were prepared for the study with educational material. Then, in a driving simulator, participants drove with PDA in driving scenarios built for the purpose of this study. The scenarios depicted driving situations in which automation could suspend depending on environmental conditions. These situations were selected by experts in the automotive industry because of their representativeness of the current functioning of driving automation systems. The use cases were bent roads, erased road markings, traffic jams, and foggy areas. Over a 3-week period, the participants performed three driving sessions, the first and last of which were considered pre-tests and post-tests, and the four intermediate sessions as training. During the study, participants’ driving behavior and visual fixations were measured. Their mental models, trust in automation, and workload were rated. The analysis plan consisted in comparing the measures of participants between the pre-tests and post-tests, depending on their interface condition, and separately for each use case.

### 1.4. Research objectives

This study aimed to assess to what extent the prolonged exposition to a Multimodal Interface with an indicator of Limits of Automation (MILA) addresses the goals proposed by Carsten and Martens (2019), considering the learning effect regarding automation and the interface. The tested goals were to stimulate an appropriate level of attention and intervention, to induce accurate mode awareness, and to induce appropriate trust in the system. The goals of Carsten and Martens were turned into a general hypothesis and divided into an operational hypothesis: (1) a MILA interface stimulates a more appropriate level of attention than a Visual Basic Interface (VBI), which would result in more gaze fixations on the instrument’s cluster before the suspension of driving automation systems and more important mental workload for MILA than VBI during the first uses but a decrease after several driving sessions; (2) MILA induces a more accurate mode awareness than VBI, which would result in more precise mental models in shorter periods of time for MILA than for VBI and better control of the vehicle when driving automation systems suspended; and (3) MILA induces a more important trust in automation than VBI.

## 2. Method

### 2.1. Participants

The sample was composed of 40 volunteers (15 women) aged 39–65 years (*M* = 53.34, *SD* = 6.83). This age group was selected because its constituents potentially belong to a socio-professional category whose financial capacity allows them to acquire a vehicle equipped with PDA. They were recruited *via* the panelist Eurosyn. It was required to be able to drive without glasses, to hold a valid driving license for at least 3 years, to drive several times a week, to have experienced cruise control at least once, and to have a positive attitude toward automation (evaluated on a response scale). If volunteers met these requirements, they were tested on their crystallized and fluid intelligence, and their visual acuity. The cognitive abilities start to decline quite early in age (i.e., approximately 20–30 years) and vary from one individual to another (Salthouse, 2009). By measuring fluid and crystallized intelligence, we ensured that the participants were representative of the general population and capable of using automated systems. The crystallized intelligence was assessed with the WAIS-IV’s Vocabulary test. The goal of the test is to define concepts and objects. It consists of 30 items of progressively increasing complexity (e.g., the first item is “peaceful,” and the last item is “castigate”). A correct answer scores 2 points, a correct but ambiguous answer scores 1 point, and a wrong answer scores 0 points. The maximum possible score is 57 points. Three consecutive zeros require the test to be stopped. Seven volunteers were excluded because they failed to define three consecutive concepts. The included participants succeeded in defining correctly around half of the concepts (*M* = 31.8; *SD* = 6.03). Fluid intelligence was assessed with the WAIS-IV’s Cancellation test. Volunteers had to cross out targets among distractors in a limited time. The score considered the speed of execution and the number of correct and incorrect responses (*M* = 15.98; *SD* = 12.84). Then, the participants took a visual acuity test in which they had to read a text with small letters at 60 cm of distance. They rated their *a-priori* trust in automation by answering the question “How much trust would you place in the driving automation system?” on a response scale ranging from 0 (low) to 10 (high). The participants were randomly assigned to one of two interface conditions. The participants signed an informed consent form and were paid 150 euros for their participation. The majority of the participants had a Cruise Control (CC) in their vehicle (*n* = 27), some had an ACC (*n* = 12), and a few did not have either a CC or an ACC, but already used it (*n* = 2). In all, 16 participants reported using their CC as much as possible, 13 use it when the situation seems appropriate, 4 reported using it sometimes, and 8 never use it. The functioning of the driving automation systems presented in this study varied from conventional CC. It was, therefore, expected that previous experience with CC would not affect the handling of the PDA. Prior to the experiment, the participants were explained the principles of PDA, functioning, and situations to use it.

### 2.2. Materials

#### 2.2.1. Driving simulator

**Figure 1:**
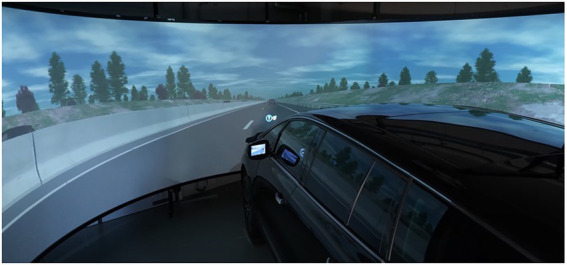
Driving simulator composed of a car with a 180° field of view. Adapted/Reproduced with permission from IRT SystemX.

A high-fidelity driving simulator was built for the purpose of the CMI Project at IRT SystemX (Palaiseau, France) where this study took place (see Figure 1). It was composed of a full-car cab with seven visual channels, providing a high-fidelity graphic resolution and realistic driving environment. Three visual channels were located in front of the vehicle providing a 180° field of view. Three visual channels were display screens showing the view from the rearview and side mirrors. The remaining visual channel was a virtual instrument’s cluster displaying the instrument cluster. The SCANeR software version 1.9 (SCANeR Software, 2020) was used to simulate the driving environment. The simulated vehicle had an automatic gearbox and two modes of automated driving could be activated (i.e., driving assistance and PDA). The steering wheel was controlled by a SensoDrive electric motor system (SENSODRIVE, 2020), which allowed to produce haptic feedback by applying torque and vibration in the steering wheel. Auditory signals in the form of earcons were emitted from the driver’s headrest.

#### 2.2.2. Driving automation systems

Driving automation systems were integrated with the simulator to simulate the driving automation systems available in today’s vehicles equipped with PDAs. A driving assistance (SEA Level 1) and the PDA (SAE Level 2) were simulated. The driving assistance was composed of only the ACC, and the PDA was composed of an LCA and an ACC. They could be activated by pressing buttons on a tactile screen on the right part of the steering wheel’s arm. They could be deactivated by pressing the same buttons or the brake pedal. Drivers could change mode independently between manual driving, driving assistance, and PDA. When switching from manual driving to driving assistance, only the ACC was activated (LCA could not be activated individually). When switching from driving assistance to PDA, the LCA was activated in addition to the ACC. When switching from PDA to driving assistance, the ACC stayed activated and the LCA was deactivated. When switching from PDA to manual driving, both the ACC and the LCA were deactivated. When switching from manual driving to PDA, both the ACC and the LCA were activated.

The following two limits of the ACC could be reached: maximum deceleration and non-detection of the followed vehicle. The maximum deceleration limit was reached when approaching a slow vehicle. The non-detection of the followed vehicle was reached when fog blocked the sensors. There were two limits of the PDA, namely, maximum lateral acceleration and non-detection of road markings. The limit of lateral acceleration could be reached when passing sharp bends and reaching an important lateral acceleration. The limit of detection of road markings was reached when the road markings were mostly or fully erased. When a limit of PDA was reached, the LCA suspended, while the ACC remained active. Once the correct condition for the LCA was present, it became active again on its own.

#### 2.2.3. Interface design

**Figure 2:**
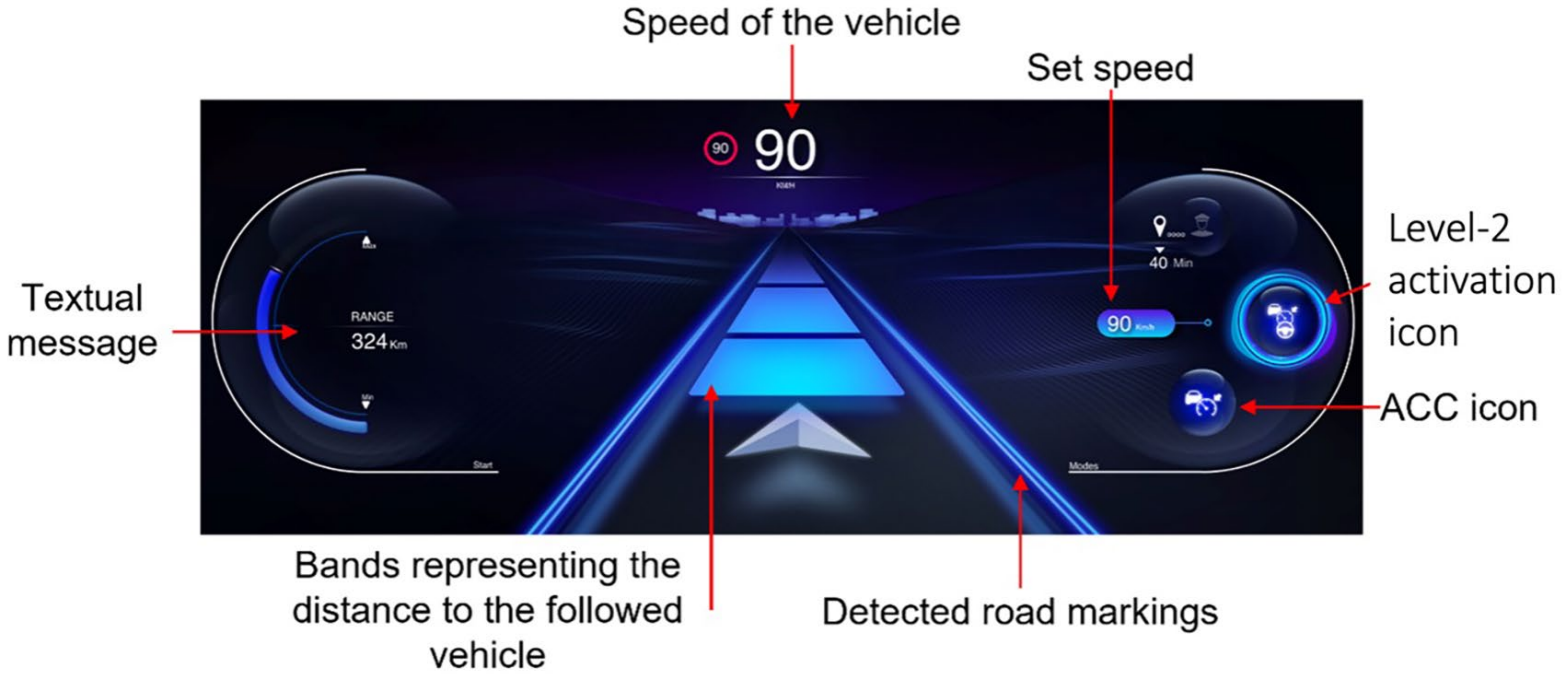
Visual representation of the instrument’s cluster of the VBI, with information that was mutual to both interfaces. The driving speed is 90 km/h. The message box on the left gives information on the remaining kilometers on the fuel tank. On the right side, the status of activation of driving automation systems and the set speed are displayed. In the center of the screen, the detected road markings and set distance of the ACC are displayed (blue). Adapted/Reproduced with permission from IRT SystemX.

The following two interfaces were compared during this study: a VBI and a MILA. These two interfaces shared similarities. They both presented the speed of the vehicle, the set speed of the ACC, the current state of the PDA and driving assistance, the detected road markings, the set distance of the ACC and a textual message area (see Figure 2). When the distance with the lead vehicle was too close with a Time To Collision (TTC) under 4 s, an auditory and visual alert was emitted. The alert was played again if TTC was below 2 s. The TTC was calculated by dividing the distance to the followed vehicle (m) by the speed of the participants’ vehicle (m/s). The two interfaces differed in the information they transmitted regarding the state and functioning of driving automation systems. The VBI only displayed the states of driving automation systems on the instrument’s cluster. It was also the case of the MILA, with the addition of an indicator of limits of automation, a haptic interface, and an auditory interface.

##### 2.2.3.1. Indicator of proximity to the limits of automation

An IPLA was presented in this interface when the PDA was activated. The IPLA informed the drivers about a risk of a transition of the state of the PDA, with the objective of allowing them to anticipate the transition and act appropriately. Its design was based on a prior study and has been improved to reduce the risk of inadequate behavior (Monsaingeon et al., 2021). It was displayed on the instrument’s cluster in order to be perceived in peripheral vision. The limits of the following two systems of the PDA were displayed: limits of LCA and limits of ACC, which also provoked a suspension of LCA. In both representations, a cloud was displayed with varying sizes depending on the proximity to the limits of automation (see Table 1). The following two degrees of limits were indicated: a moderate size and yellow cloud indicated that limits are getting closer but should not be reached, and a large red cloud indicated that limits will soon be reached. A pop-up screen appeared in the center of the instrument’s cluster when the yellow and red clouds were displayed. It represented a visual icon of the event that caused the approach of limits (e.g., representation of a bent road), a textual message of the cause of approach to the limits, and the action to perform to act appropriately.

**Table 1:** Representations of the IPLA depending on the proximity to the limits and the driving automation system.

Type of limit and degree of proximity to the limits	Representation of proximity to the limits of automation (*central textual message*)
**Limit of ACC**	
Limits at moderate proximity	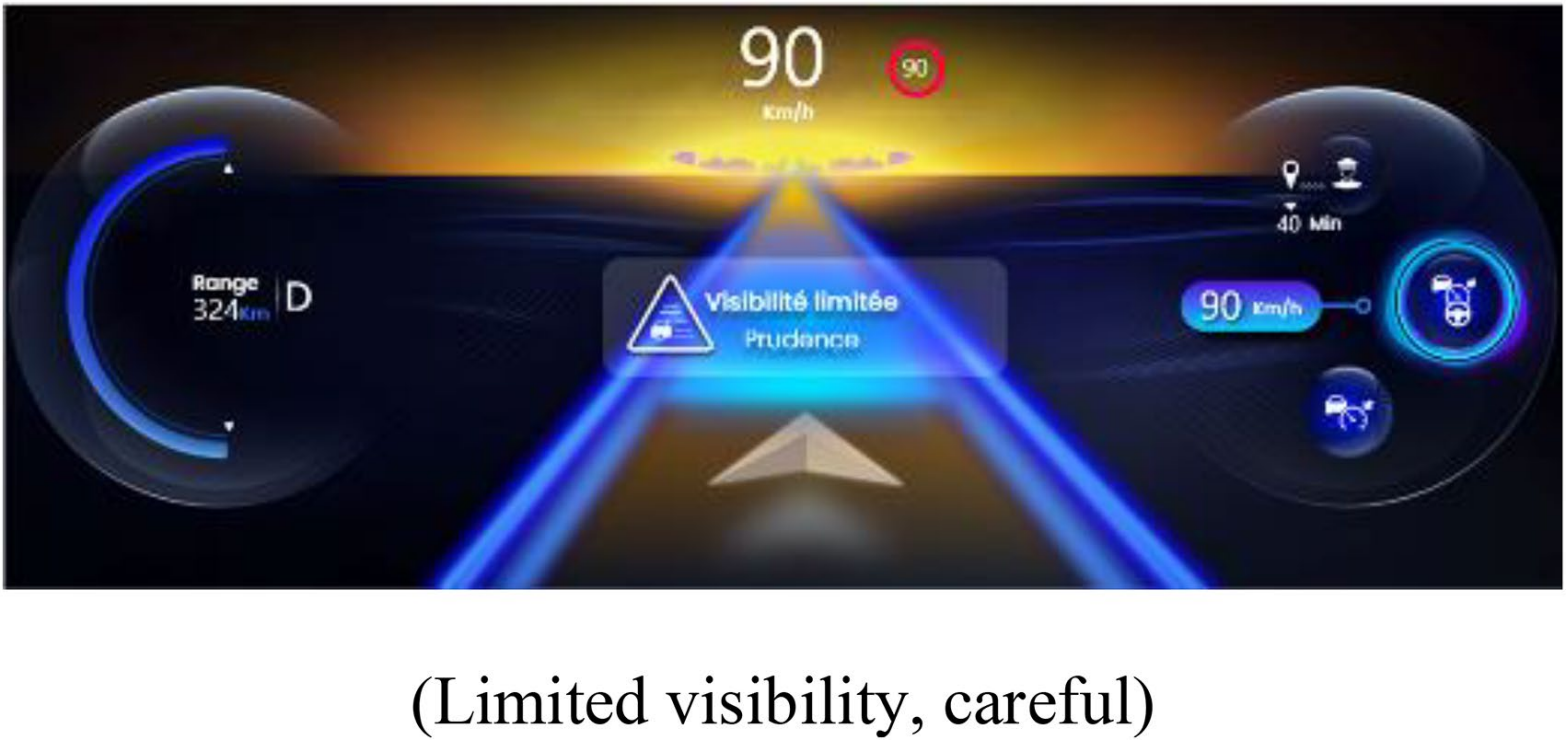
	(Limited visibility, careful)
Limits at close	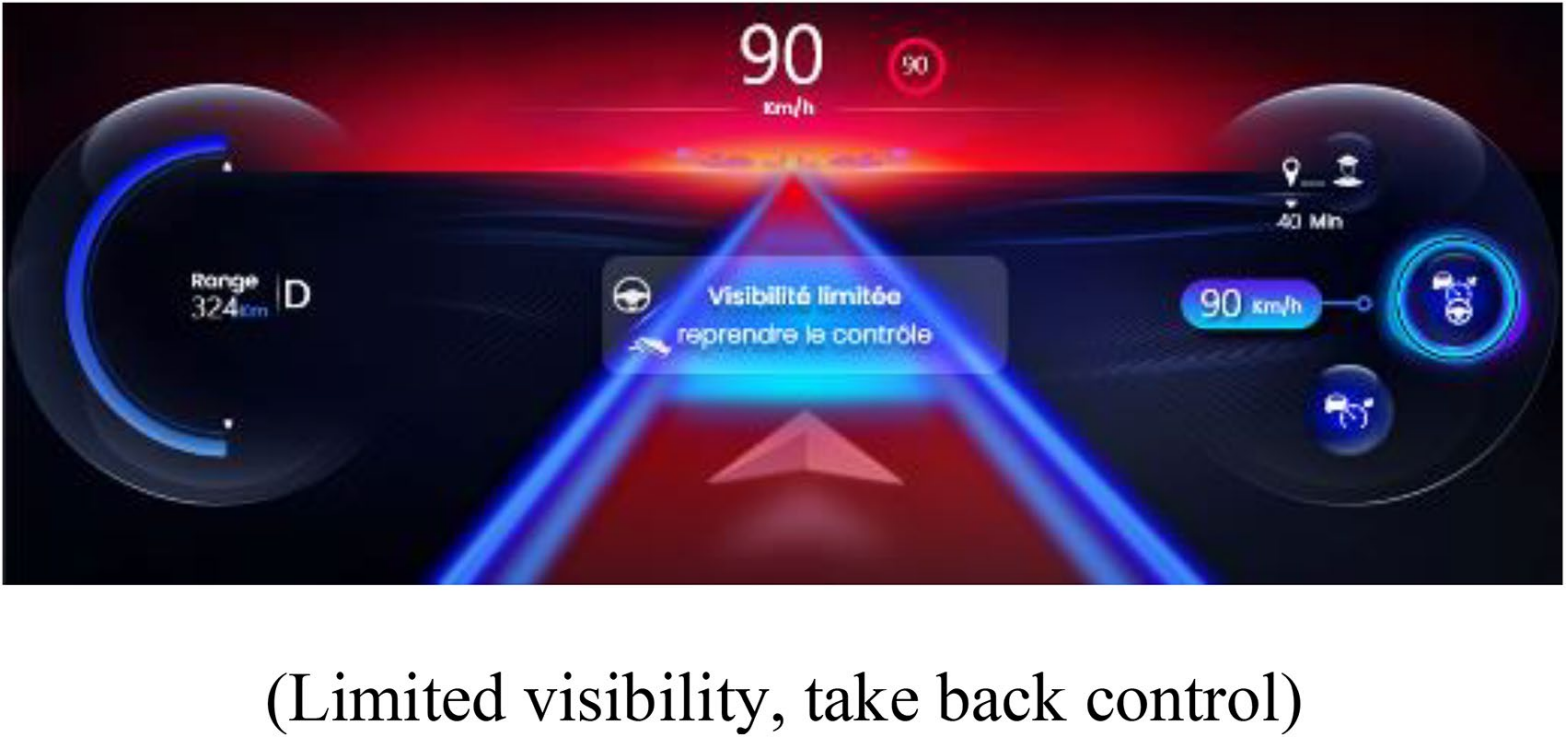
proximity	(Limited visibility, take back control)
**Limit of LCA**	
Limits at moderate proximity	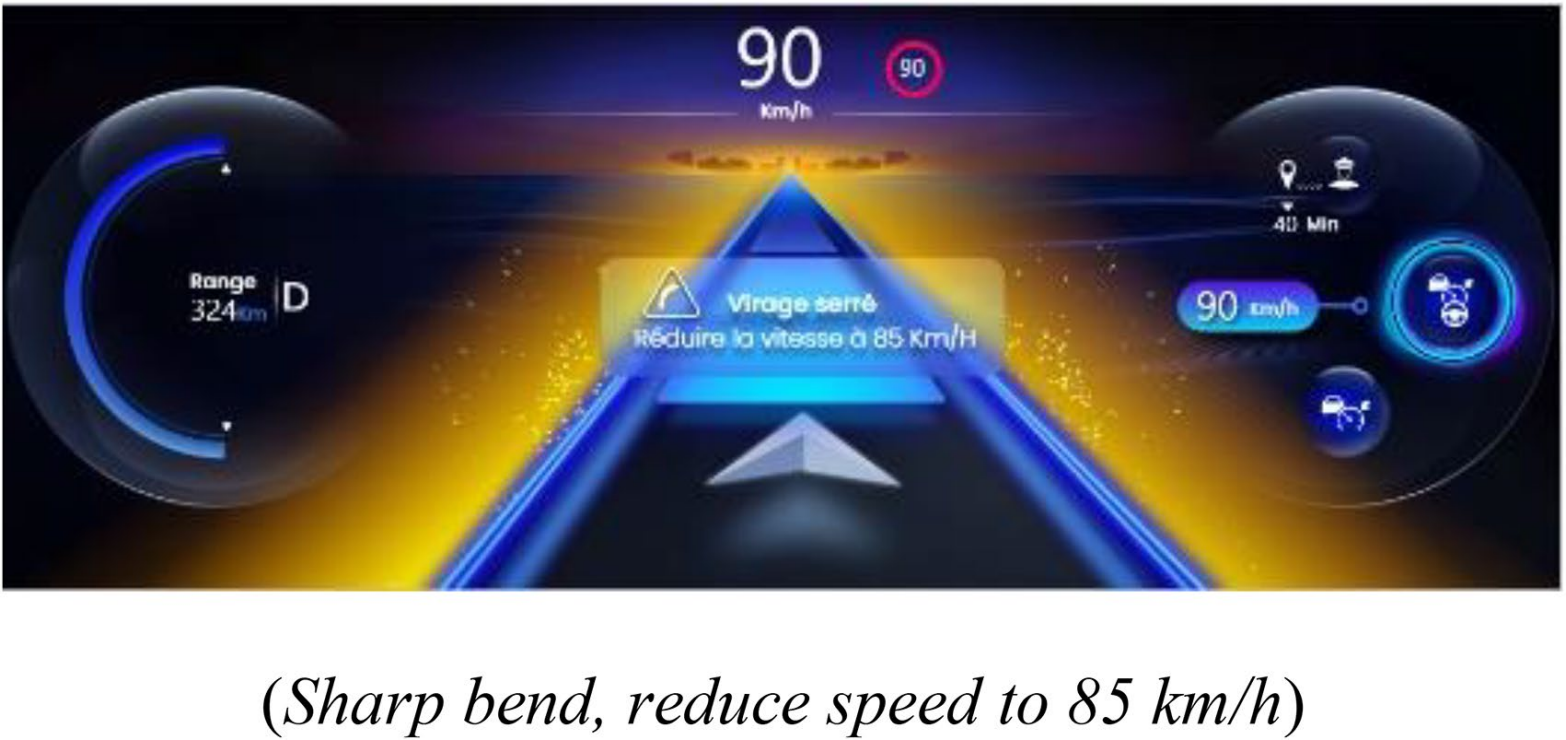
	(*Sharp bend, reduce speed to 85 km/h*)
Limits at close proximity	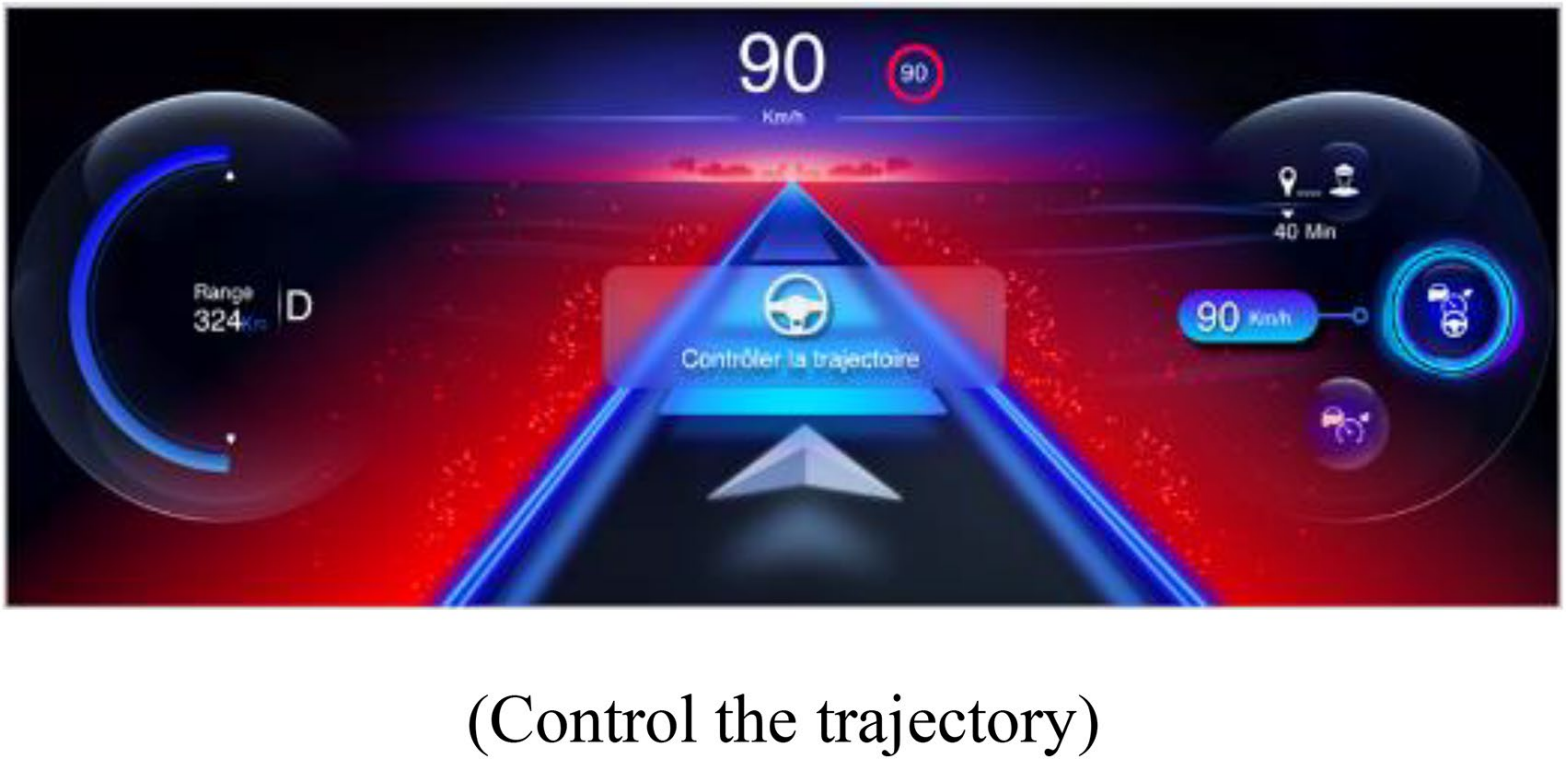
	(Control the trajectory)

The proximity to the limits of the ACC system is represented by a vertical halo (e.g., fog area). The proximity to the limits of the LCA is represented by a horizontal halo (e.g., bent road). Adapted/Reproduced with permission from IRT SystemX.

##### 2.2.3.2. Auditory interface

The auditory interface indicated when a transition of control from the system to the driver occurred. It was composed of two earcons. The efficiency of the earcons to be perceived and comprehended was evaluated in previous studies. One earcon was presented when automation transited to ACC only, meaning that it indicated a transition of control of lateral movements of the car from the system to the driver. It was composed of two descending notes. A second earcon was presented when the PDA of automation (ACC and LCA) suspended, indicating a transition of control of both lateral and longitudinal movements of the car from the system to the driver. It was composed of three descending notes. The earcons were validated through different experiments to ensure that they were perceived and comprehended.

##### 2.2.3.3. Haptic interface in the steering wheel

The following two haptic signals were transmitted through the steering wheel: kinesthetic and tactile signals. The settings of the haptic interface were tuned after the results of inter-studies. The kinesthetic signal consisted in increasing the stiffness of the steering wheel when PDA was activated. The tactile signals consisted in indicating a transition of control of the lateral movement of the car through a low-frequency vibration in the steering wheel. Two soft jerks indicated the activation of PDA, and three moderate jerks indicated a suspension of PDA. The correct perception and utility of the haptic interface were validated in previous experiments.

#### 2.2.4. Eye-tracking glasses

Our choice of eye-tracking technique took into consideration the areas fixated by drivers during the event of the scenarios. This measure allowed evaluating where the participants looked for information depending on the situation. The SMI Eye-Tracking Glasses, a pair of glasses equipped with infrared sensors to monitor eye movements (saccades, fixations, and blinks), and a frontal camera to record the field of vision were used. The eye-tracking data were recorded at a sampling frequency of 60 Hz. The glasses were connected to a mobile phone (Samsung Galaxy Note 4) that allowed us to power the glasses, calibrate the gaze measures, display the visual behavior in real time, and store the video and audio recordings. Eye-tracking data were extracted using the BeGaze version 3.7 software. We also used this software to map the fixations. This mapping consisted in associating each recorded fixation with an Area Of Interest (AOI) and was carried out by a third-party project partner. The AOIs were the instrument’s cluster, the exterior environment, and the interior environment. The BeGaze software then calculated the fixation count and duration for each AOI.

#### 2.2.5. Driving scenario

**Figure 3:**
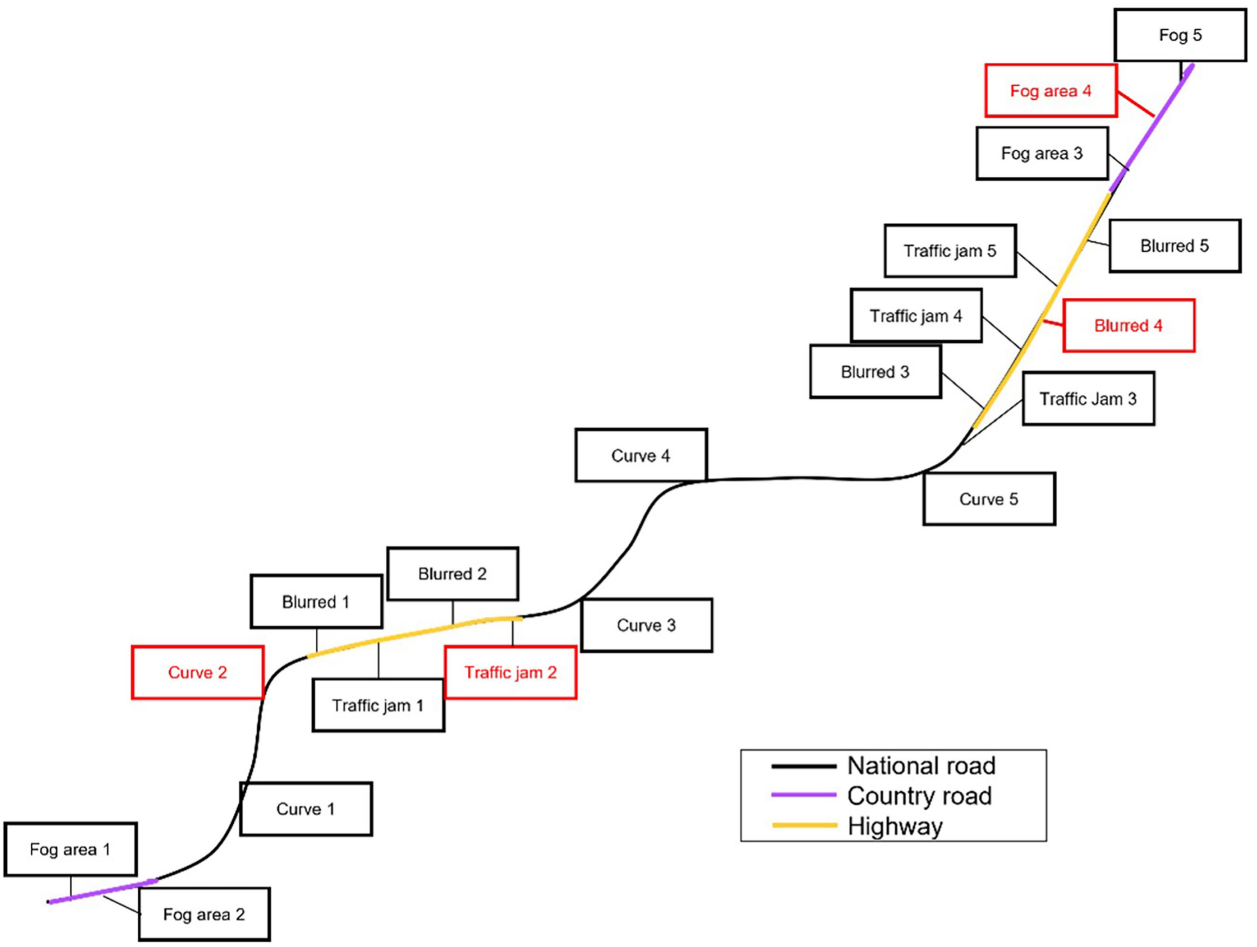
Representation of the driving scenario with the type of road and the type of event. Events marked in black did not affect driving automation systems. Events marked in red suspended driving automation systems.

The driving scenarios were created by Nervtech enterprise for this study. They depicted highways, national roads, or country roads, with moderate surrounding traffic. Between each type of road, the vehicle was teleported. The new type of road was announced on the screen of the simulator, and a black screen preceded the entry to the new type of road. A lead vehicle was always present in front of the driver. The scenarios were created not only to simulate a realistic road situation but also to control as much as possible the occurring events and replicate them for all participants. All scenarios were composed of a total of 20 events, each one separated from the other by 90 s. This duration was inspired by Beller et al. (2013). Each event lasted from 10 to 20 s, for a total duration of 30 min of driving. The events were (1) bent roads, (2) traffic jams, (3) erased road markings, and (4) foggy areas (see Figure 3). These events were chosen according to Renault Clio manual because of the possible risk to face a suspension of automation depending on the characteristics of the situation. Among the 20 events of the scenario, 16 had characteristics that allowed driving automation systems to function normally and 4 had characteristics that provoked a suspension of driving automation systems (i.e., one per type of event). These four events were randomly placed in the scenario and were identical for all participants. Each type of event was represented an equal number of times (i.e., five times by type of event).

#### 2.2.6. Tutorial

Prior to driving in the simulator, an interactive tutorial realized on Adobe Xd (version 44.1.12.5) was read by the participants. This tutorial aimed to synthesize the functioning of the driving automation systems. The tutorial was composed of four parts: (1) an explanation of what a driving automation system is, (2) the procedure to activate the driving automation systems, (3) a presentation of the limits of the driving automation systems, and (4) a summary of the tutorial with the experimenter (see Supplementary Figure 1). The description of the limits of driving automation systems consisted in presenting the following four situations that the driver could encounter during the experiment: bent roads, traffic jams, erased road markings, and areas of fog. For each situation, a description of the course of the event was given step by step, with the presented interfaces and the actions required to avoid a hazardous situation.

#### 2.2.7. Instructions

The participants were instructed to drive as much as possible with PDA driving automation systems activated during a 30-min driving scenario. They could deactivate it whenever considered necessary but had to reactivate it as soon as possible if the situation allowed it. They had to follow a white vehicle and maintain a constant distance from it and not cross it. The participants were instructed to drive at maximum legal speed.

#### 2.2.8. Familiarization scenario

A familiarization scenario was completed by the participants to initiate them to driving the simulator and automated driving systems. This scenario was a 2 × 2 straight highway without other vehicles and lasted for approximately 10 min. During this training, the experimenter, who was sited behind the participants, helped them to get used to the sensations offered by the simulator. They began by turning the steering wheel at low velocity, slowly increasing speed, and testing the brakes. Then, the experimenter guided the participants into activating and deactivating driving automation systems, changing the target speed and front vehicle distance, and informed them about the interfaces that communicated the state of driving automation systems. They witnessed the interfaces in action and were finally confronted with a situation during which driving automation systems suspended suddenly. They were warned and prepared to act accordingly.

### 2.3. Procedure

**Figure 4:**
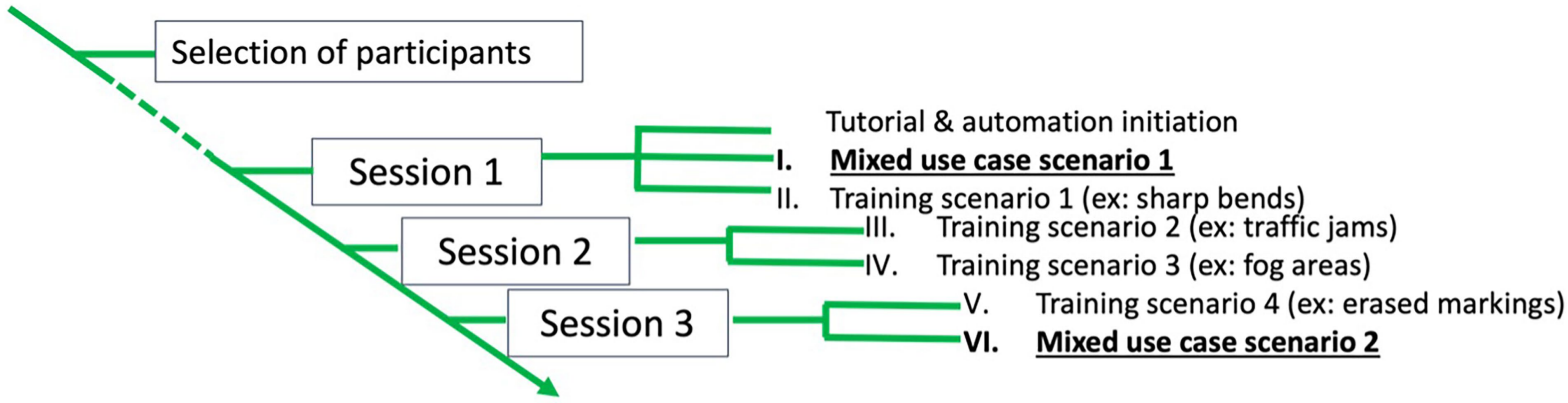
Representation of the procedure of the experiment.

Before recruitment, participants filled out questionnaires regarding their driving habits. If they were selected for the experiment, they were sent explanations regarding the experiment and the functioning of PDA-automated vehicles. The participants’ appointments were fixed for three driving sessions, one per week for 3 weeks. Each session lasted about 2 h and 30 min, resulting in a total of 7 h and 30 min of experiment per participant. At the beginning of the first session, they filled out an informed consent form. Then, the procedure of the experiment was explained. Before driving the simulator, participants were presented with a tutorial. They were instructed to read each page of the tutorial at their own pace for approximately 10 min and were free to ask questions. A familiarization scenario was completed, and the first questionnaire regarding trust toward automated vehicles was filled. The participants then began the first experimental scenario. Before each experimental driving scenario, the eye-tracking glasses were mounted on the participants and calibrated. For all participants, the first experimental scenario was a mixed situation scenario (see Figure 4). The experimenter was outside the car and did not intervene, except to deal with technical issues. Once the scenario was over, the participants were interviewed. They then filled out a mental model questionnaire and workload and trust rating scales. Four scenarios followed and were focused on specific use cases (e.g., scenarios composed only of bent roads). A Latin-square design was used to ensure that all orders of scenarios were completed with an equivalent number of repetitions. During the last session, a mixed situation scenario was performed, similar interviews to the previous scenario took place, as well as a semi-directed interview regarding the opinions of the participants toward the different interfaces. Afterward, they filled out the mental model questionnaire and workload and trust rating scales. The participants were thanked and paid for their participation.

### 2.4. Experimental design

A two-factor experimental design, mixed between and within subjects, considered two levels of interface design, namely, a baseline condition represented by VBI and a multimodal interface indicating the limits of driving automation represented by MILA, and two levels of driving scenario, namely, mixed scenario 1 and mixed scenario 2. The interface type was a between-subjects variable. The driving scenario was a within-subjects factor.

### 2.5. Measures

#### 2.5.1. Driving behavior

The driving simulation software allowed to gather vehicle parameters. The measures were similar to Langlois and Soualmi (2016) and represented the driving performance after a period of automated driving. Following Kurpiers et al. (2020) propositions, good driving performance after the suspension of driving automation systems is an indicator of accurate mode awareness. The mean distance to the center of the lane (i.e., distance between the center of the car and the center of the lane in meters) was measured after the suspension of automation in the bent road, fog areas, and erased road markings scenarios. TH was measured about 5 s before reaching the speed of a slow vehicle in traffic jams.

#### 2.5.2. Ocular movements

Gaze positions were coded on a reference image featuring eight AOIs (see Supplementary Figure 2). For each AOI, the mean fixation duration and the number of fixations were extracted during time windows of varying duration depending on the use case (see Table 2). During these time windows, the driver could have perceived the use case on the road, perceive the information on the interface, react to the use case, and return to a nominal situation. The measures during these time windows were divided into five periods: (0) preventive information (for curve only), (1) normal road before the apparition of the event, (2) information of limits of automation for MILA, (3) suspension of driving automation systems, and (4) restoration of a normal situation.

**Table 2:** Time-windows during which the measures were extracted, depending on the use case.

	Bent road	Traffic jam	Fog area	Erased markings
Before the event	30	5	10	10
After the event	40	35	30	30

The AOIs were gathered into the following two groups: on-path (on the road) and instrument’s cluster. The proportion of fixation duration on-path (on the road) was calculated by dividing the duration of fixation on-path by the total fixation duration. This measure was calculated during the period that followed the suspension of driving automation systems. The proportion of fixation on the instrument’s cluster was calculated by dividing the duration of the fixation on the instrument’s cluster by the total duration of fixation. This measure was calculated during periods that preceded the suspension of driving automation systems.

#### 2.5.3. Rating scales

##### 2.5.3.1. Mental models

A rating scale was designed to evaluate the mental model of participants regarding the functioning of driving automation systems depending on the encountered uses cases. It was inspired by the mental model rating scale for Level 2 and Level 3 vehicles of Forster et al. (2019). It was all composed of 11 points response scales, ranging from 0 (“*strongly disagree*”) to 10 (“*strongly agree*”). It included 17 items, of which 12 items covered the understanding of driving automation systems’ functioning (3 for each use case) and 5 items served as distractors. For each item of interest, one end of the rating scale was correct and the other one was incorrect (see Table 3 for detailed items of interest). Mixed linear models were used on each item of interest to evaluate the effect of each variable. The scenario and the interface were defined as fixed factors. The participant variable was defined as a random factor.

**Table 3:** Description of the affirmation of the mental model rating scale.

Use case	Affirmation	Correct answer	Type of knowledge
Curve	PDA is able to function in any type of bend.	Strongly disagree	Existence of a limit of automation
	In sharp bends, PDA becomes unavailable than reactivates itself after the bend.	Strongly agree	Presence of auto-activation
	In sharp bends, the ACC turn to suspended state.	Strongly disagree	Which driving automation system suspends
Erased road markings	When road markings are completely or very faded, the ACC and becomes unavailable, then reactivates itself.	Strongly disagree	Presence of auto-activation
	When the road markings are completely erased, PDA will ask you to take over the steering wheel.	Strongly agree	Existence of a limit of automation
	When the road markings are removed, the ACC suspends.	Strongly disagree	Which driving automation system suspends
Traffic jams	The ACC is able to brake to match the speed of the vehicle being followed, regardless of the speed of the vehicle being followed.	Strongly disagree	Existence of a limit of automation
	The ACC is suspended when braking is too important for the system.	Strongly agree	Which driving automation system suspends
	When an important braking occurs, the ACC is suspended.	Strongly agree	Which driving automation system suspends
Fog areas	The PDA is able to operate regardless of fog density.	Strongly disagree	Existence of a limit of automation
	The ACC is suspended when the fog is too dense, then reactivates itself.	Strongly disagree	Presence of auto-activation
	When the fog is too dense, the PDA suspends.	Strongly agree	Existence of a limit of automation

##### 2.5.3.2. Raw task load indeX

A French version of the Raw Task Load index (RTLX; Cegarra and Morgado, 2009) workload rating scale was completed after the first and last mixed scenarios. This rating scale consists in evaluating the workload in six dimensions (i.e., mental demand, physical demand, temporal demand, effort, performance, and frustration). The participants evaluated the workload of the driving task on a Likert-style rating scale, ranging from 0 (“*low*”) to 10 (“*high*”) for each dimension. The total workload index was calculated by summing up the ratings of each dimension.

##### 2.5.3.3. Trust in automation

To control that participants’ trust in automation before usage was not different between the interfaces groups, they rated their trust in automation on a response scale ranging from 1 (“*Not at all*”) to 10 (“*Totally trust*”) by answering the question “*How much trust would you place in the Highway and Traffic Jam Assist?.*” The difference of trust before usage was compared between the two groups with the Mann–Whitney *U*-test, with the normality of residues not being respected (*p* < 0.05). The participants reported their trust in automation after usage by rating their degree of agreement to the affirmation “*I trusted the Highway and Traffic Jam Assist during this scenario*” on a response scale ranging from 1 (“*Not at all*”) to 10 (“*Totally*”). This rating scale was completed after each driving scenario. The mean ratings of trust in automation were calculated for mixed scenario 1 and mixed scenario 2 for each group of interfaces. The progression of trust between mixed 1 and mixed 2 was assessed by subtracting the ratings of the two scenarios. The difference in progression between the two interfaces’ groups was compared with the Mann–Whitney *U*-test, with the normality of residues not being respected (*p* < 0.05).

### 2.6. Analysis

The measures of driving performance, ocular behavior, and mental workload were analyzed with mixed linear models. Measures of driving performance, visual fixation, and mental models were analyzed separately for each use case. They were presented separately for each use case. The following variables were integrated as fixed factors in the model: scenario (mixed scenario 1 vs. mixed scenario 2) and interface (VBI vs. MILA). The interactions between these factors were also integrated. The participant factor was integrated as a random factor. Bonferroni’s *post-hoc* tests were carried out when interactions were significant.

## 3. Results

### 3.1. Driving performance after suspension of driving automation systems

The driving performance was analyzed and described separately for each type of use cases (see Table 4 for summary). In the bent road use cases, the mixed linear model analysis revealed a significant effect of the scenario *F*(1,39) = 12.56, *p* = 0.001 on the mean central lane deviation, with moderate effect size (Cohen’s *d* = −0.70). The participants deviated less from the center of the lane during mixed scenario 2 (*M* = 0.46 m; *SD* = 0.45) than during mixed scenario 1 (*M* = 0.63 m; *SD* = 0.46). This result suggests that the participants of the two interface groups improved their correction of the trajectory of the vehicle after the suspension of driving automation systems. No effect of the interface and no interaction were found significant. During the fog use cases, a main effect of the interface was found on the mean central lane deviation *F*(1,38) = 8.25, *p* = 0.007, *d* = −0.65. The participants of the MILA interface (*M* = 0.43 m; *SD* = 0.25) deviated less than participants of the VBI group (*M* = 0.29 m; *SD* = 0.16). This result suggests that independently of the scenario, drivers of the MILA group had better control of the trajectory of the vehicle than drivers of the VBI group after the suspension of driving automation systems. No effect of the scenario and no interaction were found significant (*p* > 0.05). On roads where lane markings were completely erased, a significant effect of scenario was found *F*(1,39) = 10.17, *p* = 0.003, *d* = −0.57 on the mean central lane deviation. The participants deviated less during mixed scenario 2 (*M* = 1.07 m; *SD* = 0.56) than during mixed scenario 1 (*M* = 1.42 m; *SD* = 0.63). This result suggests that drivers of the two interface groups had better control of the trajectory of the vehicle after the suspension of automation during mixed scenario 2 compared to mixed scenario 1. The effect of the interface was not significant, and neither was the interaction in this use case (*p* > 0.05). In the traffic jams use cases, no effect of the scenario and interface and no interaction were found significant on the TH (*p* > 0.05).

**Table 4:** Descriptive statistics [mean (SD)] of the driving performance measures depending on the use case, scenario, and interface condition.

Use case (metric and unit)	Scenario	Interface condition	
		MILA	VBI
Bent road (mean central distance in meters)	Mixed scenario 1	1.12 (0.58)	0.81 (0.36)
	Mixed scenario 2	0.64 (0.47)	0.65 (0.39)
Traffic jams (minimal time headway in seconds)	Mixed scenario 1	2.61 (0.84)	2.20 (1.33)
	Mixed scenario 2	2.25 (0.57)	2.20 (0.69)
Fog area (mean central distance in meters)	Mixed scenario 1	0.30 (0.19)	0.43 (0.35)
	Mixed scenario 2	0.29 (0.14)	0.43 (0.14)
Erased road markings (mean central distance in meters)	Mixed scenario 1	1.48 (0.69)	1.36 (0.56)
	Mixed scenario 2	1.01 (0.57)	1.14 (0.56)

### 3.2. Eye-tracking measures

Table 5 details the descriptive statistics of fixation proportion depending on the experimental conditions and the use cases. The ratio between the total duration of visual fixation on all AIO and the duration of visual fixation of the instrument’s cluster is reported. In the bent road use cases, the mixed linear model revealed a significant effect of the interface *F*(1,39) = 11.01, *p* = 0.001, *d* = 0.76 on the proportion of fixation on the instrument’s cluster. The participants with the MILA (*M* = 0.10; *SD* = 0.15) looked more at the instrument’s cluster before a suspension of driving automation systems compared with the participants of the VBI (*M* = 0.02; *SD* = 0.04). No effect of the scenario and no interaction were revealed for this use case. This result suggests that IPLA captured the drivers’ visual attention longer than VBI before bends in which the automation suspended. In the erased marking scenario, the mixed linear model revealed a significant effect of the interface *F*(1,39) = 6.09, *p* = 0.018, *d* = 0.59. The participants with the MILA (*M* = 0.13; *SD* = 0.14) looked more at the instrument’s cluster before a suspension of PDA driving automation systems compared with the participants of the VBI (*M* = 0.05; *SD* = 0.14). No effect of the scenario and no interaction were revealed for this use cases. This result suggests that IPLA captured the drivers’ visual attention longer than VBI before areas where the erased road markings would cause the automation to suspend. In the traffic jam and fog area use cases, no effect of the interface and scenario and no interaction were found significant (*p* > 0.05).

**Table 5:** Descriptive statistics [mean (SD)] of the proportion of visual fixation on the instrument’s cluster before the suspension of automation depending on the use case, scenario, and interface condition.

Use case	Scenario	Interface condition	
		MILA	VBI
Bent road	Mixed scenario 1	0.42 (0.36)	0.09 (0.16)
	Mixed scenario 2	0.50 (0.32)	0.08 (0.16)
Traffic jams	Mixed scenario 1	0.29 (0.19)	0.35 (0.22)
	Mixed scenario 2	0.32 (0.25)	0.27 (0.15)
Fog area	Mixed scenario 1	0.08 (0.11)	0.08 (0.15)
	Mixed scenario 2	0.05 (0.08)	0.06 (0.12)
Erased road markings	Mixed scenario 1	0.15 (0.17)	0.06 (0.19)
	Mixed scenario 2	0.10 (0.08)	0.04 (0.06)

The reported numbers refer to the ratio between the total duration of visual fixation on all AIO and the duration of visual fixation on the instrument’s cluster.

### 3.3. Mental model rating scale

Results of the mixed linear models are reported for the questions regarding each use cases that yield significant effects (see Table 6 for a summary of descriptive statistics). For the bent road use cases and the affirmation “*The PDA of automation is able to function in any type of bend,”* a significant effect of the interface was observed *F*(1,39) = 5.11, *p* = 0.029, *d* = 0.60. The participants of the MILA group answered better (*M* = 6.24; *SD* = 2.98) than the participants of the VBI group (*M* = 4.30; *SD* = 3.50). A main effect of the scenario was also found for this question *F* (1, 39) = 5.82, *p* = 0.021, *d* = 0.39. The participants had a more accurate mental model after mixed scenario 2 (*M* = 5.93; *SD* = 3.58) than after mixed scenario 1 (*M* = 4.66; *SD* = 3.05). No interaction was found significant (*p* > 0.05). Overall, these results suggest that the MILA interface helped drivers form an accurate mental model of the existence of a limit to the functioning of the automation in bends and that this knowledge improved over time for both interface groups.

**Table 6:** Descriptive statistics [mean (SD)] of the scores^1^ to the mental model rating scales depending on the use case, type of knowledge investigated by the question, scenario, and interface condition.

Use case	Type of knowledge	Scenario	Interface condition	
			MILA	VBI
Bent road	Existence of a limit of automation	Mixed scenario 1	5.48 (2.68)	3.80 (3.24)
		Mixed scenario 2	7.00 (3.13)	4.80 (3.75)
	Which driving automation system suspends	Mixed scenario 1	4.14 (3.05)	5.85 (3.25)
		Mixed scenario 2	5.05 (4.06)	6.00 (4.06)
	Presence of auto-activation	Mixed scenario 1	6.43 (2.80)	5.45 (3.24)
		Mixed scenario 2	6.29 (3.51)	4.65 (3.39)
Traffic jams	Existence of a limit of automation	Mixed scenario 1	3.57 (3.40)	1.25 (1.80)
		Mixed scenario 2	3.59 (3.67)	2.75 (3.14)
	Which driving automation system suspends	Mixed scenario 1	7.33 (2.52)	6.60 (3.36)
		Mixed scenario 2	7.95 (2.48)	8.45 (2.44)
	Which driving automation system suspends	Mixed scenario 1	8.24 (2.53)	6.55 (3.66)
		Mixed scenario 2	7.10 (3.27)	7.95 (3.28)
Fog area	Existence of a limit of automation	Mixed scenario 1	6.76 (2.84)	5.55 (3.49)
		Mixed scenario 2	7.57 (3.33)	7.75 (3.06)
	Existence of a limit of automation	Mixed scenario 1	7.10 (3.71)	5.90 (3.71)
		Mixed scenario 2	7.38 (3.68)	8.60 (1.96)
	Presence of auto-activation	Mixed scenario 1	4.71 (3.33)	5.35 (3.57)
		Mixed scenario 2	4.62 (4.18)	6.15 (3.77)
Erased road markings	Existence of a limit of automation	Mixed scenario 1	7.38 (2.69)	7.50 (3.00)
		Mixed scenario 2	8.48 (2.03)	8.45 (2.33)
	Which driving automation system suspends	Mixed scenario 1	4.81 (3.50)	5.05 (3.59)
		Mixed scenario 2	5.33 (4.20)	5.95 (4.10)
	Presence of auto-activation	Mixed scenario 1	2.86 (3.20)	3.35 (3.05)
		Mixed scenario 2	2.00 (3.15)	3.25 (3.45)

1 Scores ranged from 0 to 10. Score close to 10 indicates accurate mental models.

For the traffic jams use cases, a main effect of the interface was observed for the question “*The ACC is able to brake to match the speed of the vehicle being followed, regardless of the speed of the vehicle being followed*” *F*(1,39) = 4.93, *p* = 0.032, *d* = 0.57. The participants of the MILA group had a better mental model (*M* = 3.76; *SD* = 3.50) than the participants of the VBI group (*M* = 2.00; *SD* = 2.64). This result suggests that the MILA interface helped drivers form an accurate mental model regarding the existence of a limit of automation in traffic jams. No effect of the scenario and no interaction were found significant for this question (*p* > 0.05). A significant effect of the scenario was found for the question “*The ACC is suspended when braking is too important for the system*.” *F*(1,39) = 4.30, *p* = 0.045, *d* = 0.45. The participants had a better mental model after mixed scenario 2 (*M* = 8.20; *SD* = 2.44) than after mixed scenario 1 (*M* = 6.98; *SD* = 2.95). This result suggests that the mental models of drivers in both interface groups were improved after multiple encounters with the situation.

For the fog area use cases, for the affirmation “*The PDA of automation is able to operate regardless of the fog density,*” a main effect of the scenario was found *F*(1,39) = 8.53, *p* = 0.006, *d* = 0.47. The participants had better mental models in mixed scenario 2 (*M* = 7.66; *SD* = 3.16) than in mixed scenario 1 (*M* = 6.17; *SD* = 3.19). This result suggests that the mental models of drivers in both interface groups were improved after multiple encounters with the situation. No main effect of the interface and no interaction effect were found for this question (*p* > 0.05). For the affirmation “*When the fog is too dense, the PDA of automation suspends,*” a main effect of the scenario was found *F*(1,39) = 5.04, *p* = 0.030, *d* = −0.48. The participants had a better mental model after mixed scenario 2 (*M* = 7.98; *SD* = 3.00) than after mixed scenario 1 (*M* = 6.51; *SD* = 3.21). This result suggests that mental models of drivers in both interface groups regarding which driving automation system suspends in fog were improved after multiple encounters with the situation. For all questions on erased road markings, no significant effect was observed (*p* > 0.05).

### 3.4. Trust in automation and mental workload

**Figure 5:**
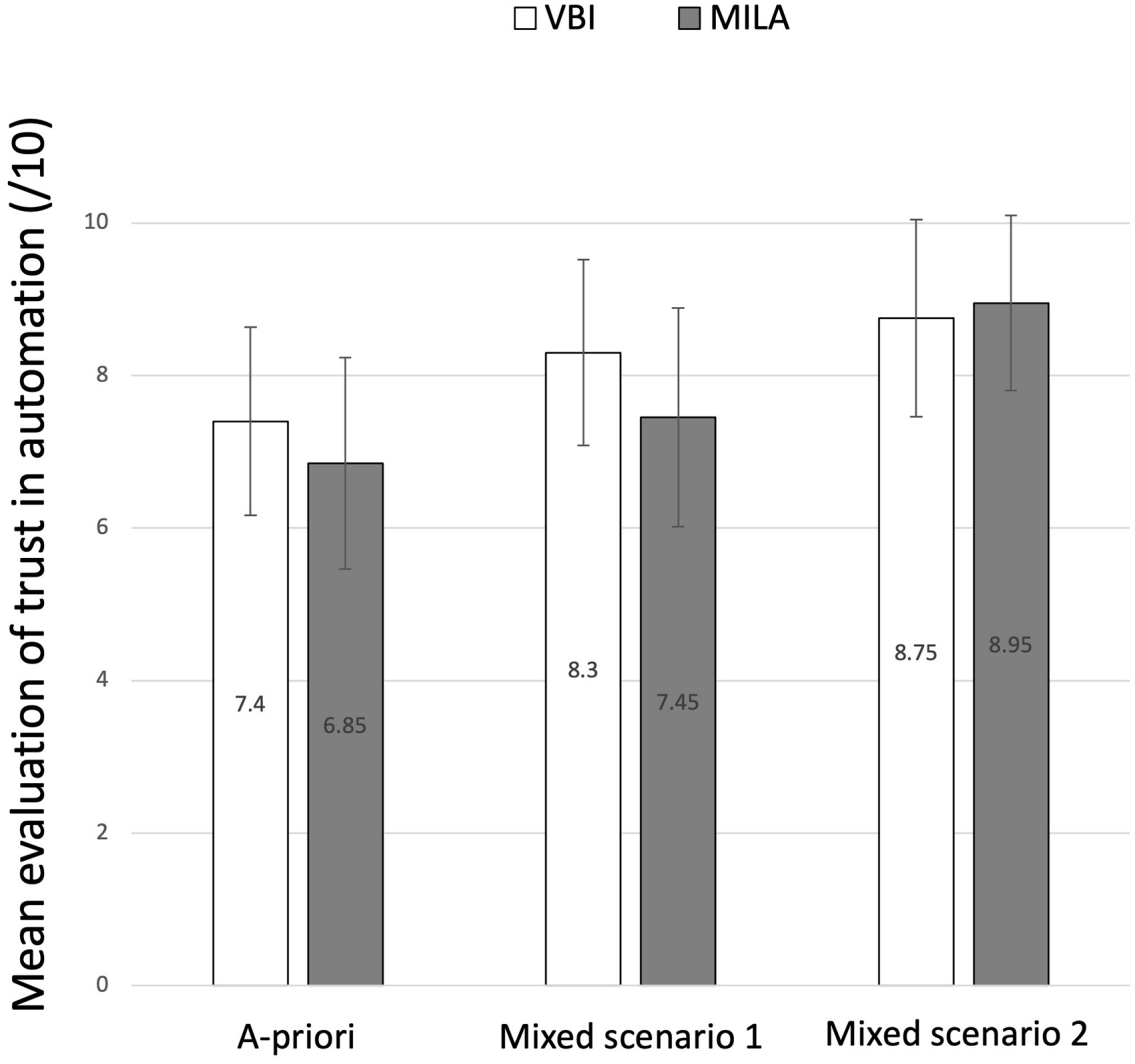
Graphic representation of the mean score to the trust scale, depending on the interface and the moment of the measure. Error bars represent the standard deviations.

**Figure 6:**
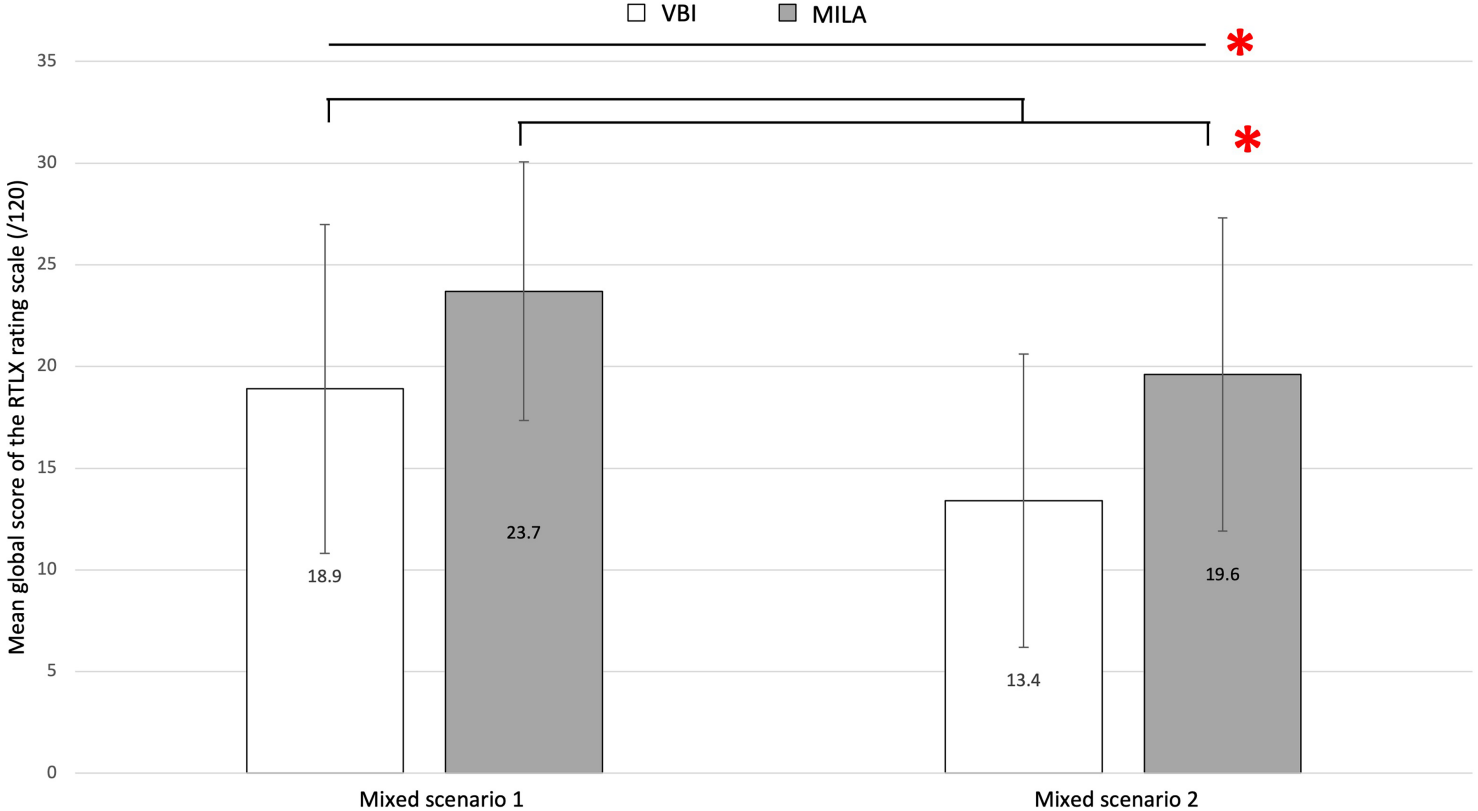
Graphic representation of the mean global RTLX scores, depending on the interface and the driving scenario. Error bars represent the standard deviations. *Significant difference (*p* < 0.05).

Nonparametric tests were carried out on measures of trust. Medians and interquartile range (IQR) are, therefore, reported. IQR represents the difference between the 75th and the 25th percentiles of the data (Upton and Cook, 1996). Regarding trust in automation before usage, the VBI group reported a slightly more important trust (*Mdn* = 7.50; *IQR* = 1.25) than the MILA group (*Mdn* = 7; *IQR* = 2.00). However, this difference was not significant (*p* > 0.05), suggesting that the two groups had a comparable *a-priori* trust before the experiment. Regarding trust after usage, a significant effect of the interface on the progression of trust across scenarios was found significant *U* = 122, *p* = 0.017, *r* = 0.42. The trust of the MILA group progressed more importantly (*Mdn* = 1; *IQR* = 2) than trust of the VBI group (*Mdn* = 0; *IQR* = 1). This result suggests that the increase in trust in automation between mixed scenario 1 and mixed scenario 2 was larger of the MILA group compared with the VBI group (see Figure 5). Regarding mental workload, the mixed model revealed a significant effect of the interface on the total score of mental workload *F*(1,38) = 7.04, *p* = 0.012, *d* = 0.74. VBI’s participants rated mental workload as lower (M = 16.2; SD = 7.71) than MILA’s participants (M = 21.6; SD = 7.65). Self-evaluated mental workload reduced significantly between mixed scenario 1 (*M* = 21.4; *SD* = 7.91) and mixed scenario 2 (*M* = 16.6; *SD* = 7.69; *F*(1,38) = 19.70, *p* < 0.001, *d* = 0.65). No interaction was found significant (*p* > 0.05). These results suggest that MILA interface was rated as causing a greater workload than VBI interface, regardless of the scenario, and that workload decreased after the mixed scenario for both interface groups (see Figure 6).

## 4. Discussion

This study aimed to evaluate to what extent a MILA stimulates an appropriate level of attention and intervention, induce accurate mode awareness, and create appropriate trust in automation, compared to a VBI interface, considering learning effects. In a driving simulator, drivers reacted to driving automation systems’ suspensions and had either one of the two interfaces. Their driving performance, ocular behavior, mental models, and self-evaluation of trust and mental workload were gathered. The following hypotheses were tested: (1) a MILA interface stimulates a more appropriate level of attention than a VBI interface, which would translate into more gaze fixations on the instrument’s cluster before the suspension of driving automation systems, heavier mental workload for MILA than VBI on first usages, but a decrease after multiple driving sessions; (2) MILA induces a more accurate mode awareness than VBI, which would translate into more precise mental models in shorter periods of time for MILA than for VBI and better driving behavior when driving automation systems suspended; and (3) MILA induces a greater trust in automation than VBI.

### 4.1. Attention stimulation

Regarding the stimulation of attention, results of ocular behavior measurements revealed that the drivers’ gaze before the suspension of automation was influenced by the interface. The participants of the MILA group gazed more importantly at the instrument’s cluster in the bent road and erased markings use cases. In these situations, the MILA interface appeared to have oriented the attention of the driver to the instrument’s cluster. This reveals that the monitoring loop of the drivers was solicited and focused on information regarding the state of the vehicle. This would be an indicator that this interface allows to put back the drivers in the loop and potentially avoid out-of-the loop phenomenon in these situations. These results are coherent with those of Monsaingeon et al. (2021), who found that an indicator of limits of automation was taken into account by drivers in their decision of action. However, these results were not observed in the traffic jam and for area use cases. This can be explained by the fact that the visual resources are heavily exploited in these situations. The fog areas demand focused vision to control the vehicle, and the traffic jams imply braking to avoid collision with the followed vehicle.

The MILA interface was more demanding than the VBI interface according to the subjective workload measurement. It appears that orienting the attention of the driver on the instrument’s cluster has an attentional cost. Both interfaces were rated as causing a very low mental workload. With both interfaces, workload decreased after the second scenario, indicating that the cost of using the driving automation systems and interfaces decreased. It was expected that the workload of the MILA interface’s group decreased more importantly than that of VBI. However, the increasing workload is not necessarily detrimental to driving performance. The Malleable Resources Theory (Young and Stanton, 2002) postulates that attentional resources are dependent on the difficulty of a task. When the task is too easy, the level of available resources decreases, causing degradation of performance. An interface that induces a more important workload might increase the difficulty of the driving task and avoid cognitive underload. Overall, the MILA appears to have induced an appropriate level of attention in the bent road and erased marking situations.

### 4.2. Mode awareness

The interface influenced the behavioral and knowledge dimensions of mode awareness. The quality of mental models was more accurate for the MILA group regarding knowledge about automation’s behavior in bent roads and traffic jams. These results regarding traffic jams are in line with Seppelt and Lee (2019) who found that continuous displays induced more accurate mental models on limits while traveling at a slow speed. However, the mental models were not better for MILA’s group regarding the erased marking and fog areas. In these situations, information on the instrument’s cluster was presented for a short period of time (3 s), on the contrary to traffic jams (8 s) and bent roads (20 s). The participants might not have been able to read the information and were not fully aware of the limits and actions to perform in these situations. This highlight that time is necessary for the drivers to integrate complex information. Moreover, an area of the instrument’s cluster was highlighted to indicate that only the LCA will suspend, and another area indicated that both the ACC and the LCA will suspend. Drivers might have not perceived the difference, explaining why questions regarding mental models on which system is suspended depending on the situation did not lead to better results. The results also revealed that knowledge regarding the limits of automation was influenced by the long-term usage of driving automation systems. These results were coherent with Forster et al. (2019) findings who showed that multiple driving sessions are necessary to form accurate mental models. However, it was expected that the mental model’s formation would be faster with the MILAs group thanks to the indicator of limits of automation, which was not observed. It appears, therefore, that the MILA interface had an immediate effect on mental models and was not influenced by multiple usages. Following the suggestions of Boelhouwer et al. (2019), in-car information impacts the formation of mental models. Our results also show that the modality and type of information is a factor in the formation of mental models. However, the extent to which mental models are correlated with driving performance is dependent on the type of situation encountered.

Regarding driving performance, participants had better driving performance after the suspension of automation in the fog area. This effect was not influenced by the learning effect, meaning that it occurred during the first interaction with automation. However, MILA’s interface did not impact driving performance in all other use cases. In contrast to Seppelt and Lee’s (2019) results, the multimodal interface did not allow to increase TTC in traffic jam situations where emergency braking was necessary. One key difference between Seppelt and Lee’s study and ours is that their auditory feedback was continuously emitted, while we used discrete auditory signals after reaching the limit of automation. Continuous auditory signals might allow to better anticipate the suspensions. Regarding the learning effect on driving performance, results revealed that drivers tended to perform better during the last scenario compared with the first scenario in bent roads and erased road markings situations. In these situations, drivers had to control the direction of the car, as only the LCA suspended while the ACC stayed activated. This finding highlights the importance of experience with automation when needed to react to surprising suspensions. Contrary to what we expected, the interface did not influence the capacity of drivers to learn to react to suspensions.

### 4.3. Relation between mental models and driving performance

Overall, the results of mental model questionnaires and driving performance indicate an asymmetry between knowledge about the system’s limit and the application of the correct driving behavior. Mental models were improved by the multimodal interface regarding the limits of automation in bent roads and erased markings, but the driving performance was not improved in these situations. Kurpiers et al. (2020) indicated a link between knowledge and behavior dimensions of mode awareness. But how the knowledge pillar interacts with the behavioral pillar? Our results suggest that improved knowledge about the system’s limit does not automatically lead to improved driving behavior. According to Rasmussen’s (1983) SRK model, knowledge and rule-and skill-based behavior are supported by different types of information. Knowledge behavior is based on symbols, while skill and rule behavior is supported by signs. Our indicator of limits of automation used both symbols and signs. Symbols (icons + text) indicated the cause of the limit of automation that would be reached and the action to perform. The sign was a halo with varying sizes and colors to indicate the proximity to the limits. Our results tend to show that the symbols were more used in the bent road and erased marking situations, leading to better mental models. It appears that symbols were prioritized to the detriment of signs, leading to better mental models, but not better driving performance. It might be possible that symbols require more attentional resources than sings and that they cannot be processed in parallel. Another argument in favor of this idea is that in the fog areas, driving behavior was better for the MILA interface group, while the gazes on the instrument’s cluster were not more important. This means that drivers did not acquire the symbols proposed on the instrument’s cluster and based their behavior on signs. Signs proposed in the IPLA might that have been perceived in peripheral vision, inducing better driving performance, but not feeding mental models.

### 4.4. Trust in automation

Trust in automation was influenced by the interface, leading to a more important increase in trust for the MILA group compared with the VBI group. This result is coherent with previous work on indicators of reliability and limits of driving automation systems (Beller et al., 2013; Helldin et al., 2013). The relationship between mental models and trust in automation was discussed by Seppelt and Lee (2019), who showed that improved mental models lead to an increase in trust. Our results confirm that suggestion. Furthermore, it appears that mental models can be improved regarding only specific aspects of driving automation systems’ functioning, and it will generally impact trust. Indicating the limits of driving automation systems can lead to small effects on mental models, but a generally positive effect on trust in driving automation systems. Interestingly, the trust of the MILA group improved even if driving behavior only slightly improved. An explanation for that would be that explicit knowledge about the driving automation systems is more important than objective behavior in trust construction. The quantity of information of MILA, evaluated as significantly more cognitively demanding compared to VBI, was also being related to improved trust. Giving more information to drivers about the automation’s functioning appears to be reassuring and could lead to a more acceptable technology.

### 4.5. Limitations

Several limitations can be reported in this study. The main limitation is that some factors might have reduced realism in the drivers’ experience. A trade-off between the ecology of situations and experimental control was necessary. Passing by 20 events at a regular pace during a 30-min drive is not a daily occurrence. The events were very controlled to ensure replicability for each participant. The only vehicles present around the drivers were those that were relevant for the event. Therefore, it missed elements like vehicles crossing or pedestrians to make the situations realistic. Moreover, the rhythm to which the event occurred could have made the driving sequences soporific, creating boredom for drivers and reducing their reaction time to suspensions of driving automation systems. The fact that drivers were equipped with eye-tracking glasses might also have reduced immersion and the realism of their reactions. We oriented toward this type of eye-tracking device to gather high-accuracy measures. Eye-tracking devices integrated into the vehicle could make the simulation more immersive but are often less accurate. A second limitation concerns the mental model questionnaires. The exact identical questionnaires were given after each scenario. Even though the relevant questions were mixed with distractive questions, the mental model of the participant might have been forged according to this question. Future studies should aim to develop mental model questionnaires that avoid repetitions.

### 4.6. Conclusion and future research

This study offers a novel insight into how interface design can improve the interaction between a human and an automated driving system. Its originality resides in the fact that novice drivers learned to use automation and that their experience was evaluated with objective and subjective measures. The participants recruited were representative of the population that buys PDA vehicles. The results revealed that multimodal interfaces with a limit of automation impact attention allocation and intervention, although this effect is context and situation dependent. It allows to improve mental models on specific knowledge but appears to have a limited impact on driving behaviors in risky events. This study highlights that improved knowledge about driving automation systems does not necessarily lead to improved driving behavior. It appears that indicators of limits of automation should integrate symbols when it aims to improve mental models and integrate signs when it aims to improve driving performance. The relationship between knowledge and behavior should be further studied to shed better light on their interaction. Even though this study takes into account the learning of drivers with 3 driving sessions separated by 1 week, some authors suggested that 2–3 weeks of daily use is necessary to master the usage of ACC (Weinberger et al., 2001). More time is maybe necessary to master the usage of ACC coupled with LCA. The answer to how mental models’ knowledge transfer to driving behavior might reside in time. Future studies should investigate the long-term usage of multimodal interfaces with limits of automation to evaluate this transfer.

## Data availability statement

The raw data supporting the conclusions of this article will be made available by the authors, without undue reservation.

## Ethics statement

Ethical review and approval was not required for the study on human participants in accordance with the local legislation and institutional requirements. The patients/participants provided their written informed consent to participate in this study.

## Author contributions

NM developed the experimental design, conducted the experiment, and wrote each version of the article. LC, SL, and CL contributed to the development of the experiment and participated in the writing of the article. All authors contributed to the article and approved the submitted version.

## Funding

This study was funded by the CMI project, a collaborative project between industrials and universities. The SystemX Technological Research Institute is a public institute that provided a significant part of the physical, financial, and human resources. The industrial collaborators Renault Group, Valeo, and Saint Gobain provided human and financial resources. The University of Bordeaux and CLLE laboratory provided human resources in the form of researchers. The authors declare that this study received funding from Renault Group. The funder was involved in the study design.

## Conflict of interest

NM and SL were employed by the company Renault Technocentre. This study was funded by the CMI project, a collaborative project between industrials and universities. The SystemX Technological Research Institute is a public institute that provided a significant part of the physical, financial, and human resources. The industrial collaborators Renault Group, Valeo, and Saint Gobain provided human and financial resources. The University of Bordeaux and CLLE laboratory provided human resources in the form of researchers. The authors declare that this study received funding from Renault Group. The funder was involved in the study design.

The remaining authors declare that the research was conducted in the absence of any commercial or financial relationships that could be construed as a potential conflict of interest.

## Publisher’s note

All claims expressed in this article are solely those of the authors and do not necessarily represent those of their affiliated organizations, or those of the publisher, the editors and the reviewers. Any product that may be evaluated in this article, or claim that may be made by its manufacturer, is not guaranteed or endorsed by the publisher.

## Footnotes

^1^For an example, see https://www.continental.com/en/press/press-releases/predictive-assistance-continental-presents-adaptive-cruise-control-with-ehorizon/
