# Supplementary figures and images for: Multimodal interface and reliability displays: Effect on attention, mode awareness, and trust in partially automated vehicles
Source: Front Psychol. 2023 Feb 28;14:1107847. doi: 10.3389/fpsyg.2023.1107847 (PMC10015246; doi:10.3389/fpsyg.2023.1107847)

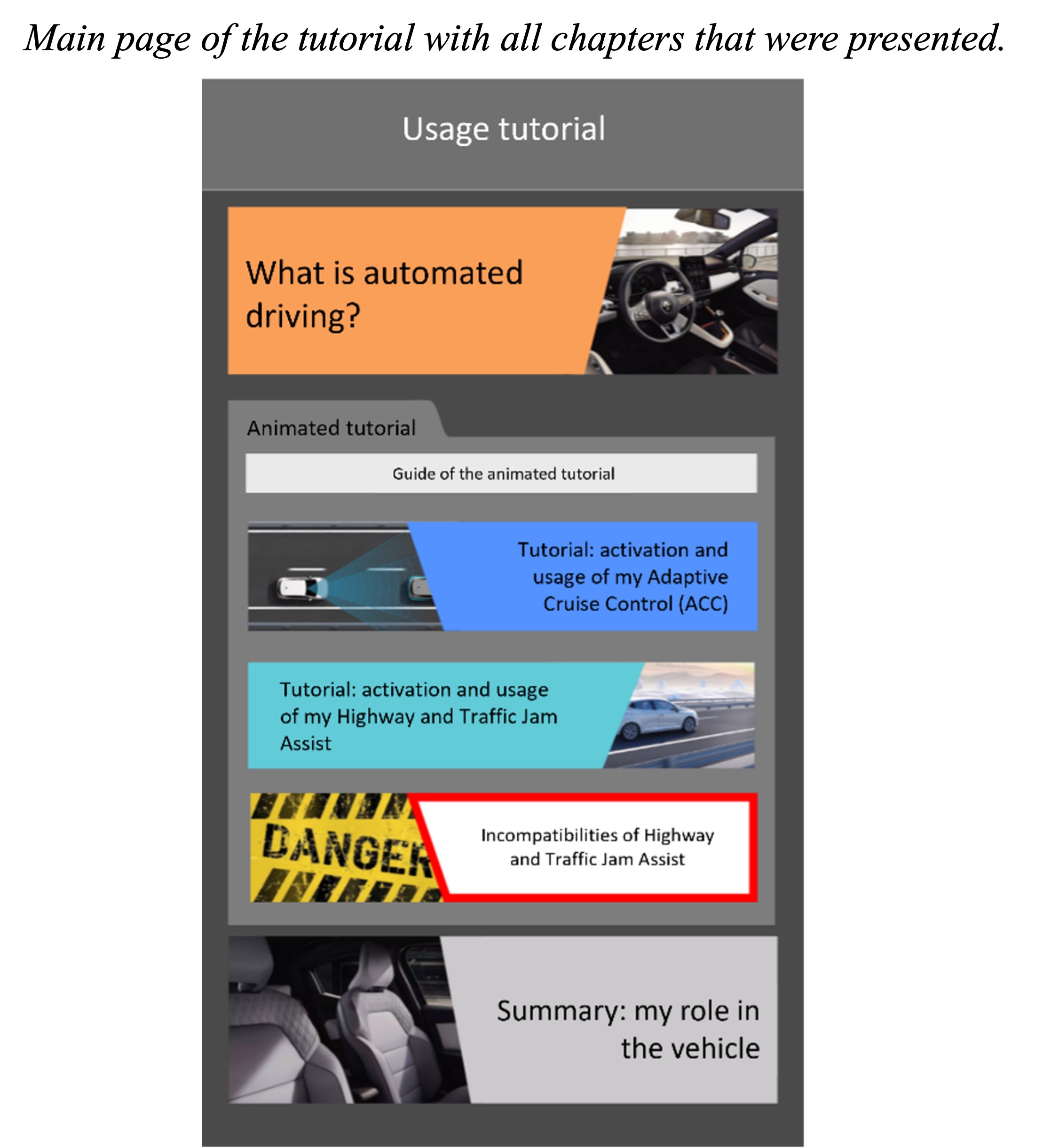

Supplement: Supplementary file 1 [file Image_1.JPEG]

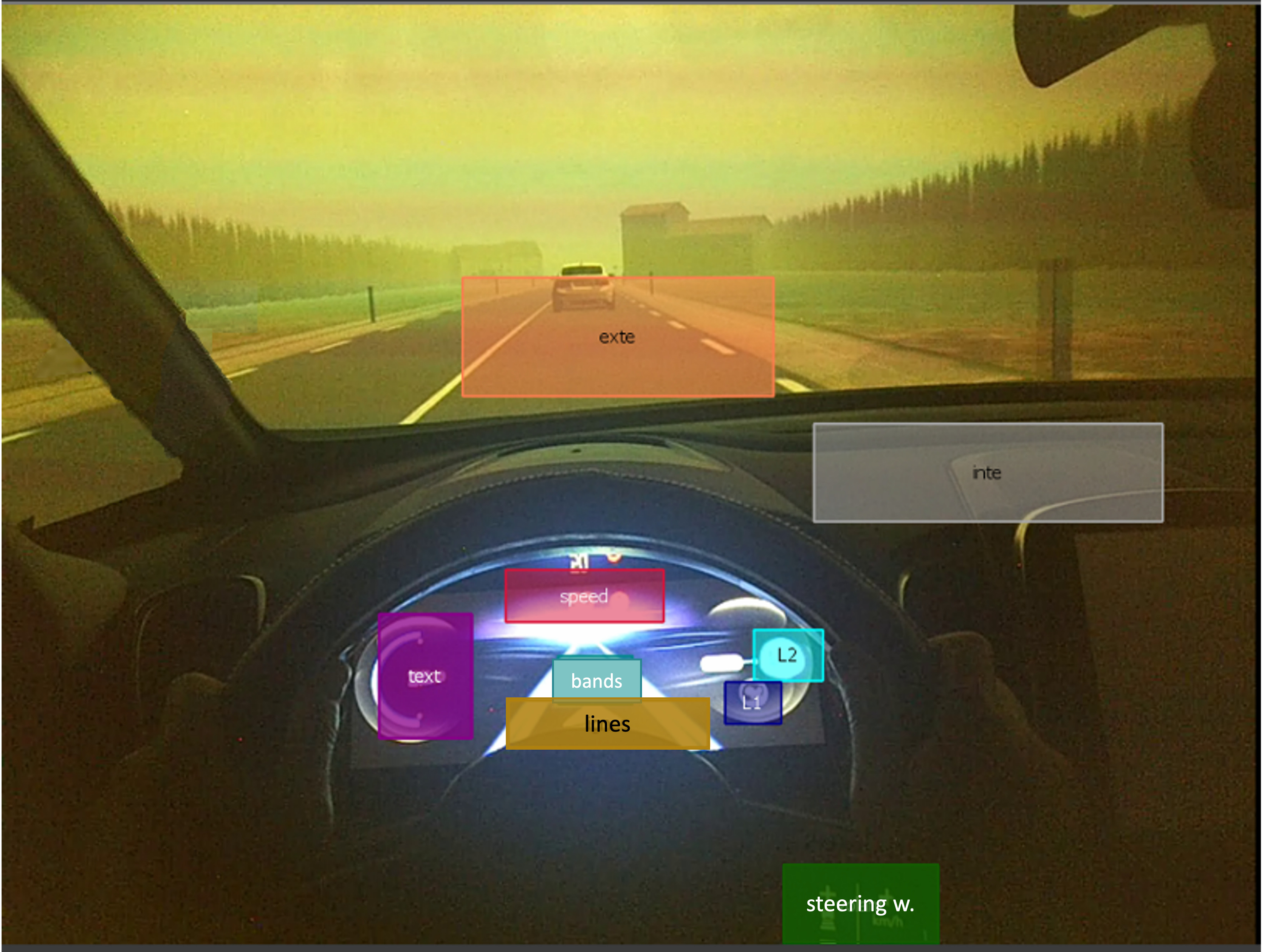

Supplement: Supplementary file 2 [file Image_2.PNG]
